# Evaluation of microRNA Expression in Patients with Herpes Zoster

**DOI:** 10.3390/v8120326

**Published:** 2016-12-02

**Authors:** Xihan Li, Ying Huang, Yucheng Zhang, Na He

**Affiliations:** 1 Department of Epidemiology, School of Public Health, The Key Laboratory of Public Health Safety of Ministry of Education, Fudan University, Shanghai 200032, China; xihanli@nju.edu.cn (X.L.); 14211020014@fudan.edu.cn (Y.Z.); 2 Department of Pain, Affiliated Drum Tower Hospital, Medical School of Nanjing University, Nanjing 210008, Jiangsu, China; huangy0808@126.com

**Keywords:** serum, microRNA, herpes-zoster, biomarker

## Abstract

Reactivated varicella-zoster virus (VZV), which lies latent in the dorsal root ganglions and cranial nerves before its reactivation, is capable of causing herpes zoster (HZ), but the specific mechanism of virus reactivation and latency remains unknown. It was proposed that circulating microRNAs (miRNAs) in body fluids could potentially indicate infection. However, the connection between herpes zoster and circulating miRNAs has not been demonstrated. In this study, 41 HZ patients without superinfection were selected. The serum miRNA levels were analyzed by TaqMan low density array (TLDA) and confirmed individually by quantitative reverse transcription PCR (RT-qPCR) analysis. Thirty-five age-matched subjects without any infectious diseases or inflammation were selected as controls. The results showed that the serum miRNA expression profiles in 41 HZ patients were different from those of control subjects. Specifically, 18 miRNAs were up-regulated and 126 were down-regulated more than two-fold in HZ patients compared with controls. The subsequent confirmation of these results by qRT-PCR, as well as receiver operating characteristic (ROC) curve analysis, revealed that six kinds of miRNAs, including miR-190b, miR-571, miR-1276, miR-1303, miR-943, and miR-661, exhibited statistically significant enhanced expression levels (more than four-fold) in HZ patients, compared with those of healthy controls and herpes simplex virus (HSV) patients. Subsequently, it is proposed that these circulating miRNAs are capable of regulating numerous pathways and some may even participate in the inflammatory response or nervous system activity. This study has initially demonstrated that the serum miRNA expression profiles in HZ patients were different from those of uninfected individuals. Additionally, these findings also suggest that six of the altered miRNA could be potentially used as biomarkers to test for latent HZ infection.

## 1. Introduction

Varicella-zoster virus (VZV) is a kind of herpes virus that is ubiquitously present in humans and causes herpes zoster (HZ), as well as chickenpox [1]. About one fifth of people worldwide could become infected with VZV at some point in their lives [2]. After the initial infection, which causes chickenpox, the VZV goes into a dormant state in the sensory ganglions and cranial nerves, and the virus is sometimes reactivated later under an immunosuppressive condition to cause HZ, also known as shingles [3]. HZ can occur at any time after chickenpox, but the incidence increases with age [4]. Cellular immunity plays an important role in regulating viral latency, as well as inhibiting reactivation [5]. In geriatric people and immunocompromised patients, such as patients suffering from end-stage renal disease, diabetes, or cancer, HZ can potentially induce bacteria super-infections which can threaten their life and CNS vasculitis [6]. In 2015, nearly 30% of zoster patients in the U.S. had hard-to-cure postherpetic neuralgia (PHN) [6]. Although antiviral drugs are successful at relieving earlier stage symptoms, these drugs cannot achieve prevention or treatment of later complications [7].

MicroRNAs (miRNAs) are a family of small, non-coding, single-stranded RNAs, which act as critical regulators of numerous diseases [8]. The number of miRNAs encoded by the human genome exceeds 500, and each kind of miRNA is capable of repressing numerous genes by binding a specific locus on their target messenger RNAs (mRNAs) [7]. MiRNAs are remarkably stable and highly resistant to degradation, particularly in comparison to mRNA [9,10]. Circulating miRNAs can become helpful biomarkers for diseases due to these special features [11,12,13]. Increasing evidence indicates that miRNAs play an essential role in maintaining cellular physiological functions, regulating interactions between host and virus, as well as inhibiting some viral replication [14].

Accordingly, our current study compares various expression profiles of serum miRNAs in HZ patients to those of healthy controls, and investigates possible physiological functions of differentially-expressed miRNAs. To our knowledge, this is the first study where the expression patterns of serum miRNAs in HZ patients have been investigated.

## 2. Materials and Methods

### 2.1. Sample Collection

A total of 76 participants were selected, 41 HZ patients and 35 healthy subjects (samples were randomly selected from healthy individuals who participated in a physical examination), from the Nanjing Drum Tower Hospital between June 2013 to December 2015. 20 serum specimens from herpes simplex virus (HSV) patients (patients undergoing clinical reactivation of HSV and confirmed by enzyme-linked immunosorbent assay, ELISA) were collected as comparisons to HZ patients. Neither the HZ patients nor the control participants were affected by secondary bacterial infection or exhibited inflammation, thereby excluding the potential of an explanatory variable. Samples were taken from the start of the acute phase for the HZ patients who exhibited clinical signs of shingles. Serum samples were delivered to the laboratory as soon as possible after collection and were stored at −80 °C within 4 h. Approval for this study was obtained from the Ethics Committee Board of Nanjing Drum Tower Hospital and all participants provided informed written consent prior to study enrolment. Demographic information was gathered by verifying electronic medical records or abstracting charts.

### 2.2. RNA Extraction

Equal volumes of serum samples from HZ patients and healthy controls were pooled into two separate pools, each consisting of 20 mL of serum. A synthetic *Caenorhabditis elegans* miRNA (cel-miR-39; 30 fmol; Takara Biotechnology Co., Dalian, China) was added into each pooled serum as an internal control before starting the isolation procedure. Total RNA were extracted from each pool using TRIzol reagent (Invitrogen, San Diego, CA, USA). Acid-phenol chloroform was briefly used to extract RNA from each serum pool and 100 μL of eluent (Ambion, Thermo-Fisher, Waltham, MA, USA) was used to elute the RNA following the manufacturer’s instructions. For the quantitative reverse transcription PCR (RT-qPCR) analysis, the reaction mixture, which included 100 μL of serum, diluted with 300 μL DEPC H_2_O, 200 μL acid phenol and chloroform, was thoroughly mixed by vortexing and then centrifuged at room temperature for 15 min. After the phases were separated, the water phase was collected and mixed with 1.5 volumes of isopropyl alcohol and 0.1 volume of 3 mol/L sodium acetate. The mixture was then incubated at −20 °C for one hour. After centrifugation at 16,000× g, 4 °C for 20 min, the obtained RNA pellet was washed with 750 mL/L ethanol and then dried at room temperature for 10 min. The dried RNA was resuspended in RNase-free H_2_O and stored at −80 °C until analysis.

### 2.3. Analysis of microRNA (miRNA) Profiles by TaqMan Low Density Array (TLDA)

TaqMan low density array (TLDA; Version 3.0; Applied Biosystems, Foster City, CA, USA) analysis was used to determine the miRNA profiles. The A and B cards were used to analyze each sample and a total of 768 miRNAs were measured in duplicate assays, including negative and internal controls. Briefly, the reaction system contained 3 μL of extracted RNA and 4.5 μL of reverse transcription (RT) mixture, which included 100 mmol dTTPs with dNTPs, 10× Megaplex RT Primer, 10× RT Buffer, 20 U/μL RNase inhibitor, 50 U/μL MultiScribe Reverse Transcriptase, 25 mmol MgCl_2_ and DEPC H_2_O. The TLDA sensitivity was improved by performing a pre-amplification step, using the Megaplex PreAmp Primer Pools A + B as well as the TaqMan PreAmp Mastermix, following the reverse transcription of the RNA. The Megaplex RT solution was diluted 150 times with H_2_O, and 450 μL of the diluted solution were mixed with an equal volume of TaqMan 2× Universal PCR Master Mix. Next, a 100 μL volume of the mixed sample and master solution of each Megaplex pool was added into the array, which was then centrifuged and mechanically sealed with the sealing equipment supplied by Applied Biosystems. A 7900 HT Fast Real-Time PCR System (Applied Biosystems) was used to perform the quantitative RT-PCR analysis using a specific cycling condition. The SDS software (Version 2.2; Applied Biosystems) was used to analyze the RT-PCR data and RQ Manager (Version 2.2; Applied Biosystems) was used to calculate the relative levels of serum miRNA. The threshold cycle (CT) values above 40 were set as undetectable. The expression level of each miRNA was normalized to cel-miR-39, and was calculated using the ΔCT method.

### 2.4. Confirming and Quantifying Candidate miRNAs through Real-Time qRT-PCR

TaqMan (Applied Biosystems) qRT-PCR analysis was used to quantify serum miRNAs. Probes, PCR primers, and RT stem loop primer were used to perform the assays. The TaqMan miRNA Reverse Transcription Kit, as well as stem loop primers (both from Applied Biosystems) specific to miRNA, were used to perform reverse transcription reactions. The scaled-down reaction system with a volume of 5 μL contained 1.67 μL of extracted RNA. The PCR reaction mixture was first incubated in a 7900 HT Fast Real-Time PCR System at 95 °C for 10 min and then maintained at 95 °C for 15 s through forty cycles, and finally cooled down to 60 °C for 60 s; the final volume was 10 μL. The reaction system contained 4.5 μL of cDNA template solution diluted at the ratio of 1/15, 0.5 μL of TaqMan miRNA Assay primer and 5 μL of TaqMan Universal PCR Master Mix (Applied Biosystems). Triplicate tests were conducted for each sample. The CT was set as the number of fractional cycles, while the fluorescent intensity exceeded the set value. Default setting values were used to assign the baseline and analyze the data.

### 2.5. Analysis of Target Genes

Target Scan (Version 7.1) was used to predict genes targeted by candidate miRNAs [15]. The Database for Annotation, Visualization and Integrated Discovery (DAVID; Version 6.8) platform was used to analyze the functions of the identified target genes and the diverse signaling pathways in which they participate [16].

### 2.6. Luciferase Assay

The entire human BCL2L1 3′ UTR segments were amplified by PCR, using human genomic DNA as a template. The PCR products were cloned into the SpeI and HindIII (Takara Biotechnology Co., Dalian, China) sites of the multiple cloning regions in pMIR-reporter plasmids (Ambion, Thermo-Fisher, Waltham, MA, USA). Insertion was confirmed by sequencing. For luciferase reporter assays, 0.2 μg of firefly luciferase reporter plasmid, 0.1 μg of β-galactosidase expression vector (Ambion), and equal amounts (20 pmol) of control RNA, mimic miR-1276, or mutant mimic miR-1276 (Takara Biotechnology Co.) were transfected into HEK293T cells in 24-well plates. The β-galactosidase vector was used as a transfection control. At 24 h post-transfection, cells were analyzed using a luciferase assay kit (Promega, Madison, WI, USA).

### 2.7. Statistical Analysis

We used the SPSS statistical software (Version 18.0; IBM Corp., Armonk, NY, USA) to perform all of the above analyses. When a *p*-value was below 0.05, it was considered highly significant. We then constructed the receiver operating characteristic (ROC) curves in order to determine the specificity, as well as sensitivity, of every miRNA individually, and in combination with other miRNAs for predicting HZ. Additionally, no participant data was missing.

## 3. Results

### 3.1. Clinical Characteristics of the Participants

A total of 41 HZ patients and 35 healthy adults, as well as 20 HSV patients, participated in this study. There were no significant differences in gender or age distribution between the HZ group and the control group (*p* > 0.05). Skin lesions were most frequently observed on the dermatomes which were dominated by C2 to L5 sensory nerve branches. The patients’ characteristics are listed in Table 1.

### 3.2. Using TLDA to Analyze the Expression Profiles of Serum miRNAs

Candidate miRNAs exhibiting varying expression profiles during HZ infection were identified using TLDA analysis. Serum miRNAs expression levels in the HZ group were compared to those in the control groups. The number of serum miRNAs identified in HZ patients and healthy controls were 235 and 312, respectively, in an array that contained a total of 768 miRNAs. The requirements for the candidate miRNAs that were differentially expressed between the HZ group and the control group were the following: (1) the CT value in either group was lower than 35, so that the detected level was considered to be reliable; (2) the expression difference of a candidate miRNA between HZ group and control group was not lower than two folds. Overall, 144 miRNAs conformed to the above requirements; 126 were decreased, while 18 were increased in HZ patients compared with the control group (Table 2). Of all the candidate miRNAs, 15 displayed notable up-regulation in the HZ group, which was 3.5-fold or higher than that in the control group and, thus, were chosen for further analysis.

### 3.3. Analysis of the Serum miRNAs Expression Profiles in the Herpes Zoster (HZ) Group by quantitative reverse transcription PCR (qRT-PCR)

**Figure 1:**
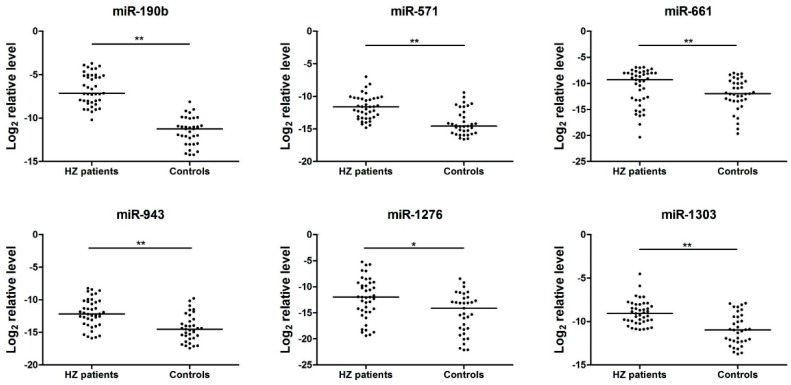
Fifteen serum microRNA (miRNA) levels in herpes zoster (HZ) patients and healthy controls were selected for verification using real-time quantitative reverse transcription PCR (qRT-PCR) in individual HZ patients (*n* = 41) and healthy controls (*n* = 35). Serum levels of miR-190b, miR-571, miR-1276, miR-1303, miR-943, and miR-661 were significantly higher in HZ patients compared with those in the control group (* *p* < 0.05, ** *p* < 0.01), while no significant differences were detected in the expression of miR-1243, miR-1238, miR-520d-3p, miR-541, miR-1225-3p, miR-605, miR-1, miR-627, and miR-1233 (*p* > 0.05). Expression levels of the serum miRNA levels were normalized to cel-miR-39 (spiked-in synthetic miRNA as an internal control).

The expression profiles of 15 candidate miRNAs that had been found to be differentially expressed in the HZ patients group compared with the healthy adults group, were verified by real-time qRT-PCR (TaqMan miRNA assays, Applied Biosystems) analysis of serum samples. As shown in Figure 1, the miR-190b, miR-571, miR-1276, miR-1303, miR-943, and miR-661 were significantly up-regulated in HZ serum samples (*p* < 0.05). Meanwhile, the expression levels of miR-1243, miR-1238, miR-520d-3p, miR-541, miR-1225-3p, miR-605, miR-1, miR-627, and miR-1233 were not significantly varied (*p* > 0.05). In order to verify the specificity of the host miRNAs for HZ infection, serum pools from HSV patients were also detected by qRT-PCR. The data of six miRNAs (miR-190b, miR-571, miR-1276, miR-1303, miR-943, and miR-661) showed significant differences with HZ patients (Supplementary Materials Figure S1).

### 3.4. Evaluation of miRNAs in Herpes Zoster (HZ) and Analysis of Variables Using Receiver Operating Characteristic (ROC) Curves

**Figure 2:**
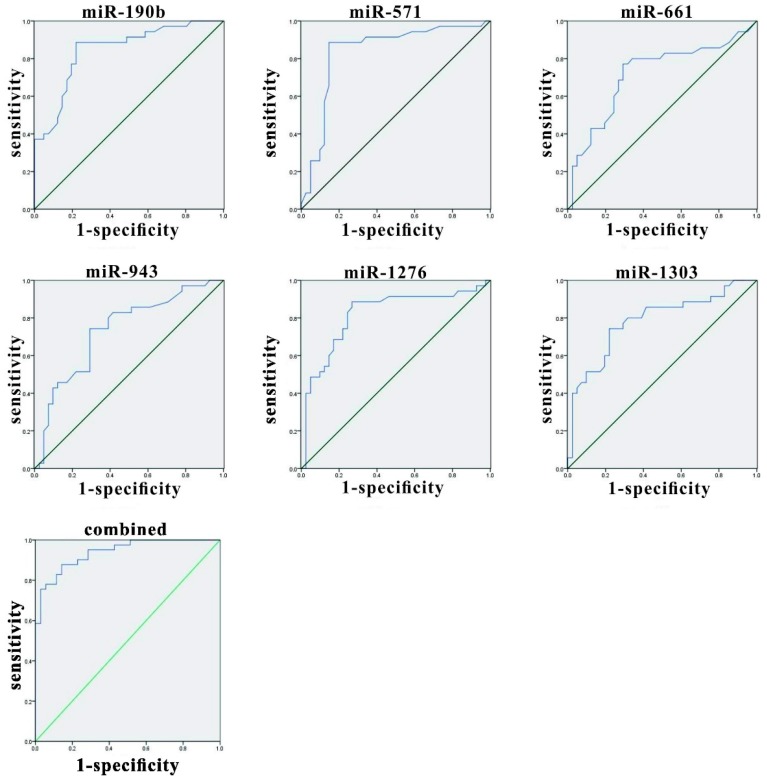
Receiver operating characteristic (ROC) curves of differentially expressed miRNAs between HZ patients and healthy controls. ROC curves of miR-190b, miR-571, miR-1276, miR-1303, miR-943, and miR-661 showed a moderate distinguishing efficiency. The combination of the six miRNAs showed a slightly higher area under the ROC curve (AUC) value of 0.939.

The effectiveness values of candidate miRNAs were evaluated by performing ROC curve analyses. The expression levels of these circulating miRNAs were notably higher in the HZ group than in the control group. The indicator variable analysis and statistical analysis using ROC curves are shown in Figure 2 and Table 3. The area under the ROC curve (AUC) of miR-190b, miR-571, miR-1276, miR-1303, miR-943, and miR-661 were 0.845 (95% CI 0.756–0.934), 0.837 (95% CI 0.737–0.936), 0.716 (95% CI 0.595–0.837), 0.733 (95% CI 0.618–0.847), 0.818 (95% CI 0.716–0.921), 0.784 (95% CI 0.678–0.890), respectively, which indicated medium distinguishing effects of these miRNAs. Multiple logistic regression analyses were conducted on these six candidate miRNAs and the AUC value obtained from the ROC curve was 0.939 (95% CI 0.890–0.987), which indicated that these miRNAs could distinguish HZ and healthy samples with high success.

### 3.5. Target Gene Prediction

Human miRNAs could exert a measurable impact on the replication of virus, the limitation of antiviral responses, the inhibition of apoptosis and the promotion of cellular growth [17]. Additionally, miRNAs have been linked to inflammatory and immune responses during bacterial infection [18]. Target Scan (Version 7.1) was used to predict target genes of miR-190b, miR-571, miR-1276, miR-1303, miR-943, and miR-661, in order to determine their potential physiological functions. Gene ontology (GO) analysis revealed that some of the identified target genes participate in the development of the immune and nervous systems (Table 4). For instance, miR-190b [19] and miR-1303 [20] regulate the expression of neuronal growth regulator 1 (NEGR1) [21], which belongs to an adhesion proteins family and has an important role in neurite outgrowth during the process of neuron development. It is also possible that regulation of this process is probably related to neuropathic pain resulting from HZ, however the specific mechanism remains unknown. It could also be inferred that this was probably due to inflammation resulting from HZ, though the exact mechanism remains to be elucidated.

**Figure 3:**
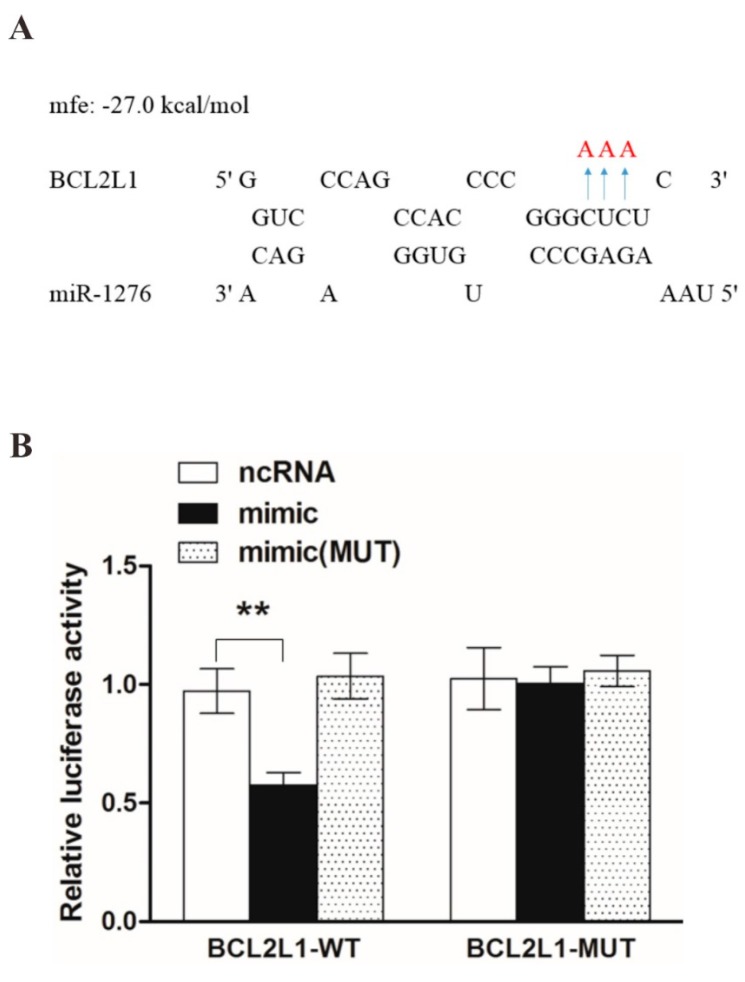
MiR-1276 targeting the 3′UTR of BCL2L1 mRNA. (**A**) The predicted binding site for miR-1276 in BCL2L1 3′UTR. In 3′UTR mutant, replaced nucleotide (red) was indicated by the arrows; and (**B**) luciferase activity in HEK293T cells transfected with plasmid encoding wild-type (WT) or mutated (MUT) 3′UTR of human BCL2L1 plus control RNA, mimic miR-1276, or mimic miR-1276 (M); (** *p* < 0.01).

To validate our targets prediction, the binding site prediction and luciferase assay were used to test the BCL2L1 as a potential target of miR-1276 preliminarily. To test whether miR-1276 regulates BCL2L1 expression, the entire BCL2L1 3′UTRs of human was sub-cloned immediately downstream of the firefly luciferase open reading frame (ORF). Additionally, we generated constructs with three nucleotide mutations in the ‘seeding’ sequence of the 3′UTR of PCBP2 (Figure 3A). We observed that miR-1276 mimic significantly down-regulated the expression of firefly luciferase fused to the BCL2L1 wild-type 3′UTR, while the mutated miR-1276 mimic did not (Figure 3B). In the reciprocal experiment, in which we transfected HEK293T cells with the firefly luciferase vector fused to the mutated BCL2L1 3′UTR, neither miR-1276 mimic nor the mutant miR-1276 mimic had an effect on the luciferase activity (Figure 3B). These results demonstrated that miR-1276 was able to directly target the sequences in the 3′UTR of BCL2L1 mRNA.

## 4. Discussion

VZV is a member of the herpes viruses group that is ubiquitously present in humans and initially causes chickenpox, and may lead to HZ [1]. About one fifth of people worldwide could become infected with the VZV virus at some point in their lives [2]. Following the primary infection, VZV lies latent in the dorsal root ganglions and cranial nerves before being reactivated years later under immunosuppressive conditions to cause HZ [3]. HZ involves relatively mild symptoms in healthy young individuals while elderly people are a high-risk population for complications [4]. It has been demonstrated that antiviral drugs are effective at alleviating earlier stage symptoms. However, the prevention and treatment of later complications cannot be achieved with them. Little is known about how VZV is reactivated from latency, but miRNAs are considered to exert a possible effect on the interaction between immunity and latency of the VZV infection. Microbial infections lead to differences in the host miRNAs expression profile, and it can have a significant influence on the study for those diseases [22]. Host miRNAs can impact viral replication and pathogenesis through various pathways.

To our knowledge, this study is the first to evaluate the expression levels of miRNAs in HZ patients. The results of this study indicated that the average serum levels of miR-190b, miR-571, miR-1276, miR-1303, miR-943, and miR-661, which were calculated by ROC curve analysis, were notably higher in HZ patients compared to those in healthy subjects. It has been previously confirmed that miR-190b exerts an important effect on the viral replication by targeting MTMR6 [23], which acts as a key regulatory factor of the immune and inflammatory responses. The up-regulation of miR-190b is driven by viral replication and not by the immune/inflammatory responses to viral infection [24]. It was initially revealed in this study that the expression level of miR-190b was higher in HZ patients compared to healthy participants. MiR-1276 was proposed as a central mediator of the NF-κB pathway by targeting BMP2 and CASP9, which act as crucial regulators of immunity and inflammation [25]. In this study, we demonstrated that miR-1276 was able to directly target the sequences in the 3′UTR of BCL2L1 mRNA, which acted as a regulator of cytokines during virus infection [26]. It could be inferred that a host may increase the miR-1276 expression to resistance the VZV infection, though the exact mechanism remains to be elucidated. MiR-571 [27] and miR-943 [28] have also been reported to play a putative role in different cell compartments involved in the fibrogenesis and inflammation processes during liver cirrhosis pathogenesis. This finding could explain the liver injury resulting from HZ infection. It has also been suggested that miR-1303 could be related to the regulation of neuroblastoma tumorigenesis and metastasis [29]. Thus, it is possible that miR-1303 is related to the complications of the nervous system which are associated with HZ.

In this study, it was discovered that several miRNAs are differentially expressed in HZ patients compared to healthy controls by TLDA analysis. The results of this study suggest that the serum miRNAs which exhibited differential expression profiles in HZ patients could be used for diagnosing this disease. Additionally, it was shown by gene ontology that the predicted target genes of the candidate miRNAs were related to the development of immune system, as well as the nervous system. The physiological mechanisms of differentially expressed miRNAs and their potential use as biomarkers for diagnosing HZ still need to be further investigated. There are still a few shortcomings. Firstly, the number of participants in this study was too small to draw definitive conclusions; secondly, only parts of the dysregulated miRNAs were investigated in this study, and biomarkers with higher efficiency could be identified by testing other miRNAs groups; and thirdly, only the serum samples from HSV patients and healthy people were used as controls to identify the specific miRNAs of HZ patients. If more controls, such as HCMV patients, measles virus patients, and influenza virus patients were used in this study, the specific miRNAs identified for HZ patients would be more precise. In the future, investigations with a larger population will be needed to verify the conclusions.

## Figures and Tables

**Table 1:** Patient characteristics.

Characteristic		Herpes Zoster (HZ) Group (*n* = 41)	Healthy Control Group (*n* = 35)
**Mean age in years (range)**		58.3 (26–85)	55.9 (22–79)
**Sex (percentage)**			
	Male	23 (56.1%)	16 (45.7%)
	Female	18 (43.9%)	19 (54.3%)
	chi square test	*p = 0.367*	
**HZ localization (percentage)**			
	C2-L1	27 (65.9%)	0
	Facial nerve dermatomes	7 (17.1%)	0
	Ophthalmic nerve dermatomes	4 (9.7%)	0
	Ramsay Hunt syndrome	3 (7.3%)	0

**Table 2:** Up-regulated microRNAs (miRNAs) in HZ patients, compared with controls, using a TaqMan low density array (TLDA).

miRNA	ΔCt_HZ_	ΔCt_control_	ΔΔCt
miR-1243	7.86978	23.06114	−15.1914
miR-190b	7.273803	21.02214	−13.7483
miR-520d-3p	8.989834	22.06215	−13.0723
miR-627	8.4600315	21.14534	−12.6853
miR-541	10.102917	22.06213	−11.9592
miR-571	12.504569	23.02614	−10.5216
miR-1238	12.89762	23.16214	−10.2645
miR-1303	11.215716	21.06214	−9.84642
miR-943	13.273769	23.06799	−9.79422
miR-1225-3p	13.785332	23.40662	−9.62129
miR-1276	14.727001	23.58135	−8.85435
miR-661	11.416535	20.06211	−8.64558
miR-605	12.586351	21.06514	−8.47879
miR-1	15.524136	21.14534	−5.6212
miR-1233	6.993761	12.05311	−5.05935
miR-99a	10.33614	13.75413	−3.41799
miR-1825	14.144214	16.97566	−2.83145
miR-598	7.228752	9.272097	−2.04335

The different Ct values between two groups was calculated by the **ΔΔ**Ct method: **Δ**Ct_HZ_ = Ct_target miRNA_ − Ct_cel-miR-39_; **Δ**Ct_control_ = Ct_target miRNA_ − Ct_cel-miR-39_; **ΔΔ**Ct = **Δ**Ct_HZ_ − **Δ**Ct_control_.

**Table 3:** AUC and the asymptotic 95% CIs of the individual miRNA in ROC curves.

miRNA	AUC	SE	Asymptotic Significance	Asymptotic 95% CI	
				Lower Bound	Upper Bound
**miR-190b**	0.845	0.045	<0.001	0.756	0.934
**miR-571**	0.837	0.051	<0.001	0.737	0.936
**miR-1276**	0.716	0.062	0.001	0.595	0.837
**miR-1303**	0.733	0.058	0.001	0.618	0.847
**miR-943**	0.818	0.052	<0.001	0.716	0.921
**miR-661**	0.784	0.054	<0.001	0.678	0.890
**Combined miRNAs**	0.939	0.025	<0.001	0.890	0.987

CI, confidence interval; SE, standard error, AUC, area under the curve; ROC, receiver operating characteristic.

**Table 4:** Genes predicted to be targeted by the candidate miRNAs.

miRNAs	Go Term	Genes
**miR-190b**	Nervous system	*NEUROD1*, *NLGN1*, *NEGR1*, *NRG3*, *NAV3*
	Immune system	*MTMR6*, *BNIP3L*
**miR-571**	Nervous system	*PAINP*, *NLGN3*, *NYAP2*
	Immune system	*FASLG*, *FCAMR*, *CASP8*, *IGSF6*, *IGLL5*
**miR-1303**	Nervous system	*NEGR1*, *NXPE2*
	Immune system	*IGSF1*, *BAG2*, *CASP14*, *IGJ*
**miR-943**	Nervous system	*CDNF*, *NRP1*
	Immune system	*IL8*, *CASP10*
**miR-1276**	Nervous system	*NMB*
	Immune system	*BMP2*, *CASP9*, *BNIP2*, *ILDR1*, *BCL2L1*
**miR-661**	Nervous system	*DRAXIN*, *IGF1*, *COL4A4*, *NRCAM*, *CDK5R1*
	Immune system	*TCTA*, *IL17REL*, *IL16*, *BOK*, *SIX4*

## Supplementary Materials

The following figure is available online at www.mdpi.com/1999-4915/8/12/326/s1, Figure S1. Serum miRNA levels in 6 herpes zoster (HZ) patients and herpes simplex virus (HSV) controls were selected for verification using real-time qRT-PCR.
