# Supplementary material for: Evaluation of microRNA Expression in Patients with Herpes Zoster
Source: Viruses. 2016 Dec 2;8(12):326. doi: 10.3390/v8120326 (PMC5192387; doi:10.3390/v8120326)
Supplement: Supplementary file 1 [file viruses-08-00326-s001.pdf]

Supplementary Materials: Evaluation of microRNA Expression in Patients with Herpes Zoster

Xihan Li, Ying Huang, Yucheng Zhang, Na He

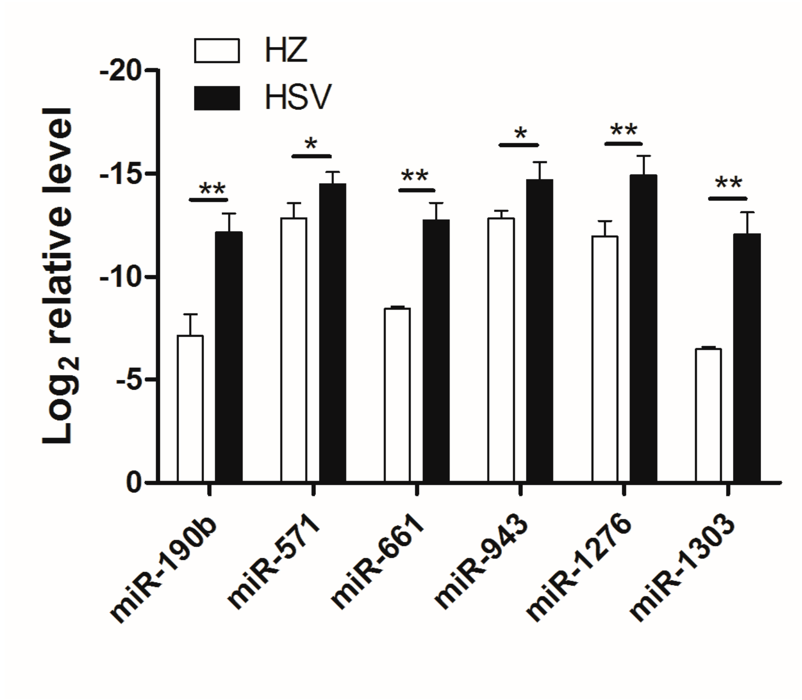

Figure S1. Serum miRNA levels in 6 herpes zoster (HZ) patients and herpes simplex virus (HSV) controls were selected for verification using real-time qRT-PCR. Serum levels of miR-190b, miR-571, miR-1276, miR-1303, miR-943 and miR-661 were significantly higher in HZ patients compared with those in the HSV group (*$p < 0.05$, **, $p < 0.01$). Expression levels of the miRNAs are normalized to cel-miR-39 (Log2 relative level).

© 2016 by the authors; licensee MDPI, Basel, Switzerland. This article is an open access article distributed under the terms and conditions of the Creative Commons Attribution (CC-BY) license (http://creativecommons.org/licenses/by/4.0/).
